# Kidney function and dementia risk in community-dwelling older adults: the Shanghai Aging Study

**DOI:** 10.1186/s13195-020-00729-9

**Published:** 2021-01-11

**Authors:** Mengjing Wang, Ding Ding, Qianhua Zhao, Wanqing Wu, Zhenxu Xiao, Xiaoniu Liang, Jianfeng Luo, Jing Chen

**Affiliations:** 1 Department of Nephrology, National Clinical Research Center for Aging and Medicine, Huashan Hospital, Fudan University, 12 Middle Wurumuqi Road, Shanghai, 200040 China; 2 National Clinical Research Center for Aging and Medicine, Huashan Hospital, Fudan University, 12 Middle Wurumuqi Road, Shanghai, 200040 China; 3 Institute of Neurology, Huashan Hospital, Fudan University, Shanghai, China; 4 Department of Biostatistics, School of Public Health, Fudan University, Shanghai, China; 5 Key Laboratory of Public Health Safety of Ministry of Education (Fudan University), Shanghai, China

**Keywords:** Glomerular filtration rate, Kidney function, Dementia, Cognitive decline, Older adults

## Abstract

**Background:**

Association between kidney dysfunction and dementia has been studied in western cohorts, but with inconsistent conclusions which may be due to the different measurements of kidney function. We aim to verify the hypothesis that lower levels of kidney function would be associated with increased risk of incident dementia in Chinese elderly.

**Methods:**

One thousand four hundred twelve dementia-free participants aged 60 years or older from the Shanghai Aging Study were enrolled and followed up for 5.3 years on average. Glomerular filtration rate (GFR) was calculated by using combined creatinine–cystatin C CKD-EPI (Chronic Kidney Disease Epidemiology Collaboration) equation. Diagnoses of incident dementia and Alzheimer’s disease (AD) were established using DSM-IV and NINCDS-ADRDA criteria based on medical, neurological, and neuropsychological examinations to each participant. Cox proportional regression was used to analyze the association of baseline GFR_crcys_ levels with incident dementia/AD, adjusting age, gender, education years, APOE-ε4, diabetes, hypertension, baseline Mini-Mental State Examination score, and proteinuria.

**Results:**

A total of 113 (8%) and 84 (7%) participants developed dementia and AD. Comparing to participants with high GFR_crcys_ (≥ 80 ml/min/1.73 m^2^), participants with low (< 67 ml/min/1.73 m^2^) and moderate GFR_crcys_ (67 ≤ GFR < 80 ml/min/1.73 m^2^) had increased risk of incident dementia with hazard ratios (HRs) of 1.87 (95% CI 1.02–3.44) and 2.19 (95% CI 1.21–3.95) after adjustment for confounders, respectively. Low (HR = 2.27 [95%CI 1.10–4.68]) and moderate (HR = 2.14 [95% CI 1.04–4.40]) GFR_crcys_ at baseline was also independently associated with incident AD after adjustments when comparing to high GFR_crcys_. The significant association between GFR_crcys_ and dementia risk was observed in female but not in male participants.

**Conclusions:**

GFR_crcys_ may be considered as a marker of an individual’s vulnerability to the increased risk of cognitive decline.

**Supplementary information:**

**Supplementary information** accompanies this paper at 10.1186/s13195-020-00729-9.

## Background

Almost 50 million people worldwide are suffering from dementia in this aging society, and this number is projected to increase to 131 million by 2050 [1]. Globally, the burden of dementia which is imposed on society is only in part reflected by the estimated $818 billion per annum, which already exceed those for any other disease [2]. Identification of risk factors of dementia is of great importance given the absence of effective treatments.

Reduced kidney function may occur as part of the aging process, and it has growing public health significance due to its association with cardiovascular outcomes, frailty, and other adverse health events [3, 4]. Moreover, kidney function decline could result in vascular injury and metabolic abnormalities which may lead to a high possibility of incident dementia [5]. A few large-sampled population-based studies have investigated the relationship between kidney dysfunction and dementia onset but reported inconsistent results [6–11]. The major heterogeneity in these studies is the different measurements of kidney function, i.e., inverse of serum creatinine (1/SCr), estimated glomerular filtration rate (GFR) based on creatinine (GFR_cr_) or cystatin-C (GFR_cys_). Another might be the different diagnoses of dementia, i.e., DSM criteria or discharge diagnosis. Additionally, almost all the evidence was from the western population. Data from the Asian population, especially Chinese, are still very limited.

The Shanghai Aging Study has collected baseline serum samples for cystatin C and creatinine measurements, in addition to the comprehensive, clinical interview for cognitive function in a community-based cohort [12]. In this study, we aim to verify the hypothesis that lower levels of kidney function would increase incident dementia risk in Chinese elderly.

## Methods

### Study site and population

The Shanghai Aging Study is a prospective cohort study to enumerate the prevalence, incidence, and risk factors for dementia and mild cognitive impairment (MCI) among older residents living in an urban community of Shanghai, China. This study recruited 3141 permanent residents aged ≥ 60 years at baseline from January 2010 to September 2011. Detailed procedure of the recruitment has been published elsewhere [12].

At baseline, 156 dementia cases were diagnosed; therefore, 2985 participants were eligible to be followed up from April 2014 to December 2016 to prospectively observe the incident dementia during the first 5-year interval. There were 1326 individuals who were lost to follow-up, including 263 deceased, 271 refused to be interviewed, 779 with whom we lost contact, and 13 were not able to cooperate with clinical interviews and neuropsychological testing. Among the 1659 participants who were successfully followed, 247 were excluded for missing data of serum cystatin C, creatine, or Apolipoprotein (APOE)-e4 at baseline (Figure S1). Our final study population consisted of 1412 participants. This study was approved by the Medical Ethics Committee of Huashan Hospital, Fudan University, Shanghai, China (approval number: HIRB2009-195). A written informed consent was obtained from all of the participants and/or their legal guardians.

### Laboratory measures and estimation of GFR

At baseline, a 10-ml spot urine sample was collected for urinalysis which detects proteinuria by using Sysmex UF-1000i analyzer (TOA Medical Electronics, Kobe, Japan). Fasting blood samples were drawn for laboratory examination.

Serum creatinine and cystatin C were determined using Roche Cobas 8000 modular analyzer Series (Roche, Inc., Mannheim, Germany) with the enzymatic assay creatinine Plus Ver.2 (Roche, Mannheim, Germany) and a particle-enhanced immunonephelometric assay (Roche, Tina-quant Cystatin C, Mannheim, Germany) on cobas c702 and cobas c501 platforms, respectively.

We calculated GFR by Chronic Kidney Disease Epidemiology Collaboration (CKD-EPI) equations. We chose the combined creatinine–cystatin C equation of CKD-EPI which performed better than only creatinine or only cystatin C equation as the primary GFR_crcys_ estimation method [13].

### Neuropsychological assessments, neurological examinations, and consensus diagnosis

A neuropsychological test battery was used to assess the cognitive function of each participant which covers the following domains: global cognition, executive function, spatial construction function, memory, language, and attention. The battery contained the (1) Mini-Mental State Examination (MMSE), (2) Conflicting Instructions Task (Go/No Go Task), (3) Stick Test, (4) Modified Common Objects Sorting Test, (5) Auditory Verbal Learning Test, (6) Modified Fuld Object Memory Evaluation, (7) Trail-making tests A and B, and (8) RMB (Chinese currency) test. All tests were administered by certified study psychometrists according to the education level of each participant and were conducted in Chinese within 90 min. Normative data and a detailed description of these tests are reported elsewhere [14, 15]. Objective impairment in cognitive domain was defined as performance 1.5 SD below the mean using the norms obtained in a pilot study which was conducted in healthy elderly living in the same community. The average age of the population used to develop the normative data with ≥ 6-year education was 71.6 years old and was 78.0 years old for those with < 6-year education. This distribution is similar with the current study cohort with an average age of 70.3 years old for participants with ≥ 6-year education and of 76.4 years old for those with < 6-year education.

The neurological examination included motor responses and reflexes for each participant. Depression symptom was determined by Center for Epidemiologic Studies Depression Scale (CESD) and was present if the CESD score ≥ 16 [16]. Neurologists also administered the Clinical Dementia Rating scales [17, 18] and activities of daily living (ADL) [19] to elicit physical activities of daily living and memory complaints.

After the clinical interview, neurologists, neuropsychologists, and neuroepidemiologists reviewed the functional, medical, neurological, psychiatric, and neuropsychological data and reached a consensus diagnosis for dementia using DSM-IV criteria [20]. Alzheimer’s disease (AD) was diagnosed by NINCDS-ADRDA criteria [21]. Cognitive function of participants was evaluated using the same neuropsychological battery and consensus diagnosis of incident dementia was conducted by the same diagnosis group using the same diagnostic criteria at both baseline and the 5-year follow-up visit.

### Measurement of confounders

At baseline, trained research nurses interviewed participants face-to-face to collect information on their demographic characteristics (gender, age, education year) and lifestyle factors, e.g., cigarette smoking. History of hypertension, diabetes, and stroke were asked and confirmed from their medical records. Besides, participants’ weight and height were measured to calculate the body mass index (BMI). DNA was extracted from blood or saliva collected from study participants. APOE genotyping was conducted by the Taqman SNP method [22]. The presence of at least one ε4 allele was considered as being APOE-ε4 positive.

### Statistical analysis

Continuous variables of participants’ characteristics were expressed as mean ± standard deviation (SD), and categorical variables were expressed as percentages or ratios. Characteristics of participants were presented stratified by baseline GFR_crcys_ tertiles (GFR_crcys_ < 67, 67 ≤ GFR_crcys_ < 80, GFR_crcys_ ≥ 80 ml/min/1.73 m^2^). These values were also decided according to the fact of GFR decline due to healthy aging of the kidney and the distribution of measured GFR in elderly population [23]. We used non-parametric trend tests to assess differences in baseline characteristics across GFR categories. The cumulative incidence curves of dementia and AD for the GFR categories were plotted using the Kaplan-Meier method, and the difference was tested using a log-rank test.

Univariate and multivariate Cox proportional hazards regression analyses were used to assess the hazard ratio (HR) and 95% confidence interval (CI) of dementia and Alzheimer’s disease associated with low/moderate GFR category, compared with the high GFR category. Confounders in the adjusted model included age, gender, education years, APOE-ε4, diabetes, hypertension, baseline MMSE score, and proteinuria. A sensitivity analysis was conducted to assess the association by excluding participants with stroke history. Stratified subgroup analyses were performed according to age (≥ 70 or,< 70 years old), gender, hypertension, diabetes, presence of APOE-ε4, serum creatinine (≥ 0.85 or < 0.85 mg/dl), and BMI (≥ 24 or < 24 kg/m^2^). Potential effect modification by the above stratified status was evaluated by significance tests of the cross-product interaction terms of the stratified status and GFR category by using the Wald test.

Data analyses were conducted using STATA MP version 13.1 (StataCorp, College Station, TX). All *P* values and 95% CIs were estimated in two-tailed tests. Differences were considered statistically significant at *P* < 0.05.

## Results

### Baseline characteristics of study participants

As shown in Table 1, among 1412 participants in this study, those with moderate GFR_crcys_ (67 ≤ GFR < 80 ml/min/1.73 m^2^) and high GFR_crcys_ (≥ 80 ml/min/1.73 m^2^) tended to be younger; had a higher prevalence of diabetes, hypertension, higher MMSE score, and diastolic blood pressure; and had a lower prevalence of stroke, lower cystatin C, creatinine, and uric acid. During a mean follow-up of 5.3 years (7484.6 person-years total), 113 (8%) participants developed dementia, and of these, 84 (6%) were diagnosed with incident AD.

**Table 1:** Baseline characteristics of 1412 participants stratified by GFR_crcys_ tertiles

Characteristics	Total *N* = 1412	GFR_crcys_ < 67 *N* = 454	67 = <GFR_crcys_ < 80 *N* = 465	GFR_crcys_ > =80 *N* = 493	*P* for trend
**Age (years)**	70.69 ± 6.85	73.49 ± 6.95	70.79 ± 6.68	68.01 ± 5.78	< 0.001
**Male (%)**	659 (46.67%)	224 (49.34%)	216 (46.45%)	219 (44.42%)	0.13
**Body mass index (kg/m**^**2**^**)**	24.83 ± 3.45	24.95 ± 3.39	24.68 ± 3.31	24.86 ± 3.63	0.35
**Education years**	11.99 ± 3.97	11.91 ± 4.36	11.90 ± 4.01	12.16 ± 3.54	0.86
**Smoking (%)**	144 (10.20%)	50 (11.01%)	52 (11.18%)	42 (8.52%)	0.20
**Comorbidities (%)**					
Diabetes	197 (13.95%)	54 (11.89%)	62 (13.33%)	81 (16.43%)	0.04
Hypertension	748 (52.97%)	267 (58.81%)	229 (49.25%)	252 (78.71%)	0.02
Stroke	180 (12.75%)	72 (15.86%)	59 (12.69%)	49 (9.94%)	0.006
Depression	31 (2.20%)	11 (2.42%)	11 (2.37%)	9 (1.83%)	0.53
**MMSE score**	28.35 ± 1.88	28.02 ± 2.27	28.37 ± 1.75	28.64 ± 1.52	< 0.001
**SBP (mmHg)**	145.67 ± 22.29	147.00 ± 22.51	145.26 ± 22.29	144.81 ± 22.07	0.13
**DBP (mmHg)**	77.81 ± 11.42	76.85 ± 11.45	77.78 ± 11.40	78.71 ± 11.37	0.04
**GFRcrcys (ml/min/1.73 m**^**2**^**)**	73.79 ± 15.63	56.20 ± 8.62	73.52 ± 3.63	90.26 ± 7.89	< 0.001
**GFRcys (ml/min/1.73 m**^**2**^**)**	71.13 ± 17.62	53.18 ± 10.02	69.64 ± 7.45	89.06 ± 11.14	< 0.001
**GFRcr (ml/min/1.73 m**^**2**^**)**	76.34 ± 14.68	60.73 ± 10.70	77.96 ± 7.73	89.18 ± 7.97	< 0.001
**Proteinuria positive (%)**	37 (2.63%)	15 (3.32%)	9 (1.94%)	13 (2.64%)	0.54
**APOE-e4 allele positive (%)**	233 (16.50%)	74 (16.30%)	78 (16.77%)	81 (16.43%)	0.96
**Laboratory variables**					
Cystatin C (mg/l)	1.05 ± 0.23	1.28 ± 0.22	1.03 ± 0.09	0.85 ± 0.10	< 0.001
Creatinine (mg/dl)	0.90 ± 0.23	1.09 ± 0.24	0.87 ± 0.14	0.75 ± 0.14	< 0.001
Uric acid (mg/dl)	5.81 ± 1.44	6.55 ± 1.56	5.67 ± 1.23	5.26 ± 1.19	< 0.001
Triglyceride (mg/dl)	156.56 ± 102.45	155.84 ± 79.50	146.51 ± 81.38	166.71 ± 133.56	0.42
Cholesterol (mg/dl)	208.55 ± 40.67	208.21 ± 41.20	208.86 ± 40.80	208.59 ± 40.13	0.92
Low-density lipoprotein cholesterol (mg/dl)	129.43 ± 35.36	128.93 ± 35.72	131.09 ± 34.70	128.32 ± 35.66	0.62
High-density lipoprotein cholesterol (mmol/l)	51.52 ± 13.03	50.56 ± 12.69	52.35 ± 13.21	51.63 ± 13.14	0.30
**Follow-up MMSE score**	26.91 ± 3.80	25.98 ± 4.91	27.05 ± 3.24	27.63 ± 2.83	< 0.001
**Incident rate of dementia, cases per 100 person years (95% CI)**	1.51 (1.26–1.82)	2.57 (2.00–3.31)	1.50 (1.09–2.08)	0.56 (0.34–0.94)	< 0.001

Values are expressed as mean ± SD or percentage, appropriately. Conversion factors for units: creatinine in mg/dl to μmol/l, 88.4; uric acid in mg/dl to mmol/l, 0.0595; triglyceride in mg/dl to mmol/l, 0.0113; cholesterol in mg/dl to mmol/l, 0.0259; LDL in mg/dl to mmol/l, 0.0259; HDL in mg/dl to mmol/l, 0.0259

*MMSE* Mini-Mental State Examination, *SBP* systolic blood pressure, *DBP* diastolic blood pressure, *APOE* apolipoprotein E, *GFRcrcys* Glomerular filtration rate estimated by CKD-EPI creatinine–cystatin C equation, *GFRcr* glomerular filtration rate estimated by CKD-EPI creatinine equation, *GFRcys* glomerular filtration rate estimated by CKD-EPI creatinine–cystatin C equation

### Incidence rate of dementia

**Fig. 1:**
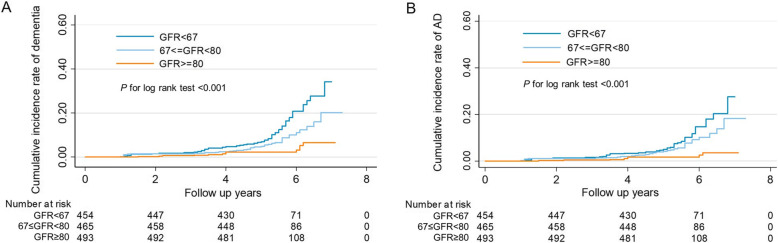
Kaplan-Meier failure curve and confidence intervals (CIs) for **a** dementia or **b** Alzheimer’s disease in three glomerular filtration rate (GFR_crcys_) tertiles

Dementia incidences were 2.57 (95% CI 2.00–3.31)/100 person-years, 1.50 (95% CI 1.09–2.08)/100 person-years, and 0.56 (95% CI 0.34–0.94)/100 person-years in groups with low, moderate, and high GFR_crcys_, respectively, while incidence rates of AD were 1.77 (95% CI 1.31–2.40)/100 person-years, 1.30 (95% CI 0.92–1.84)/100 person-years, and 0.38 (95% CI 0.20–0.70)/100 person-years in groups with low, moderate, and high GFR_crcys_, respectively. Cumulative incidence rate of dementia and AD in participants with high GFR_crcys_ was significantly lower than those with moderate and low GFR_crcys_ (log-rank test, *P* < 0.001) (Fig. 1a, b).

### Association between GFR and incident dementia

Comparing to participants with high GFR_crcys_, participants with moderate (HR = 2.77 [95% CI 1.53 to 5.05]) and low GFR_crcys_ (HR = 4.90 [95% CI 2.79 to 8.63]) had increased risks of incident dementia in the unadjusted model. The associations remained significant after adjusted for age, gender, education year, APOE-ε4, hypertension, diabetes, baseline MMSE, and proteinuria. The adjusted HR for participants with moderate GFR_crcys_ was 1.87 (95% CI 1.02–3.44) and the adjusted HR for those with low GFR_crcys_ was 2.19 (95% CI 1.21–3.95). Comparing to high GFR_crcys_, low (adjusted HR =2.14 [95% CI 1.04 to 4.40]) and moderate GFR_crcys_ (adjusted HR = 2.27 [95% CI 1.10 to 4.68]) at baseline were also independently associated with higher risk of incident AD, after adjusted for confounders (Table 2). A sensitivity analysis that excluded 180 participants with a history of stroke resulted in similar results. Low GFR_crcys_ was also associated with a higher risk of incident dementia (adjusted HR = 2.19 [95% CI 1.12 to 4.29]) and AD (adjusted HR = 2.80 [95% CI 1.19 to 6.58]) in fully adjusted models when comparing to high GFR_crcys_. Additionally, moderate GFR_crcys_ was associated with a higher risk of incident AD comparing to high GFR_crcys_ (adjusted HR = 2.47, 95% CI 1.05–5.86) (Table 2).

**Table 2:** Associations of baseline glomerular filtration rate (GFR_crcys_) estimated by the CKD-EPI equations with incidence of dementia and Alzheimer’s disease

	Dementia				Alzheimer’s disease			
	Unadjusted		Adjusted		Unadjusted		Adjusted	
	HR (95% CI)	*P*	HR (95% CI)	*P*^a^	HR (95% CI)	*P*	HR (95% CI)	*P*^a^
**All participants**								
**Tertile of GFR**_**crcys**_								
GFR_crcys_ > 80 ml/min/1.73 m^2^	Ref		Ref		Ref		Ref	
67 ≤ GFR_crcys_ < 80 ml/min/1.73 m^2^	2.77 (1.53 to 5.05)	0.001	1.87 (1.02 to 3.44)	0.04	3.60 (1.77 to 7.32)	< 0.001	2.27 (1.10 to 4.68)	0.03
GFR_crcys_ < 67 ml/min/1.73 m^2^	4.90 (2.79 to 8.63)	< 0.001	2.19 (1.21 to 3.95)	0.009	5.06 (2.54 to 10.10)	< 0.001	2.14 (1.04 to 4.40)	0.04
**Participants without stroke history**								
**Tertile of GFR**_**crcys**_								
GFR_crcys_ > 80 ml/min/1.73 m^2^	Ref		Ref		Ref		Ref	
67 ≤ GFR_crcys_ < 80 ml/min/1.73 m^2^	2.71 (1.38 to 5.33)	0.004	1.78 (0.89 to 3.55)	0.10	3.99 (1.72 to 9.26)	0.001	2.47 (1.05 to 5.86)	0.04
GFR_crcys_ < 67 ml/min/1.73 m^2^	4.59 (2.42 to 8.71)	< 0.001	2.19 (1.12 to 4.29)	0.02	6.03 (2.67 to 13.64)	< 0.001	2.80 (1.19 to 6.58)	0.02

*GFR*_*crcys*_ glomerular filtration rate estimated by the CKD-EPI creatinine–cystatin C equation, *HR* hazard ratio, *CI* confidence interval

^a^*P* adjusted for age, gender, education years, APOE-ε4 positive, diabetes, hypertension, mini-mental state examination (MMSE), and proteinuria (positive, negative)

### Subgroup analysis

**Fig. 2:**
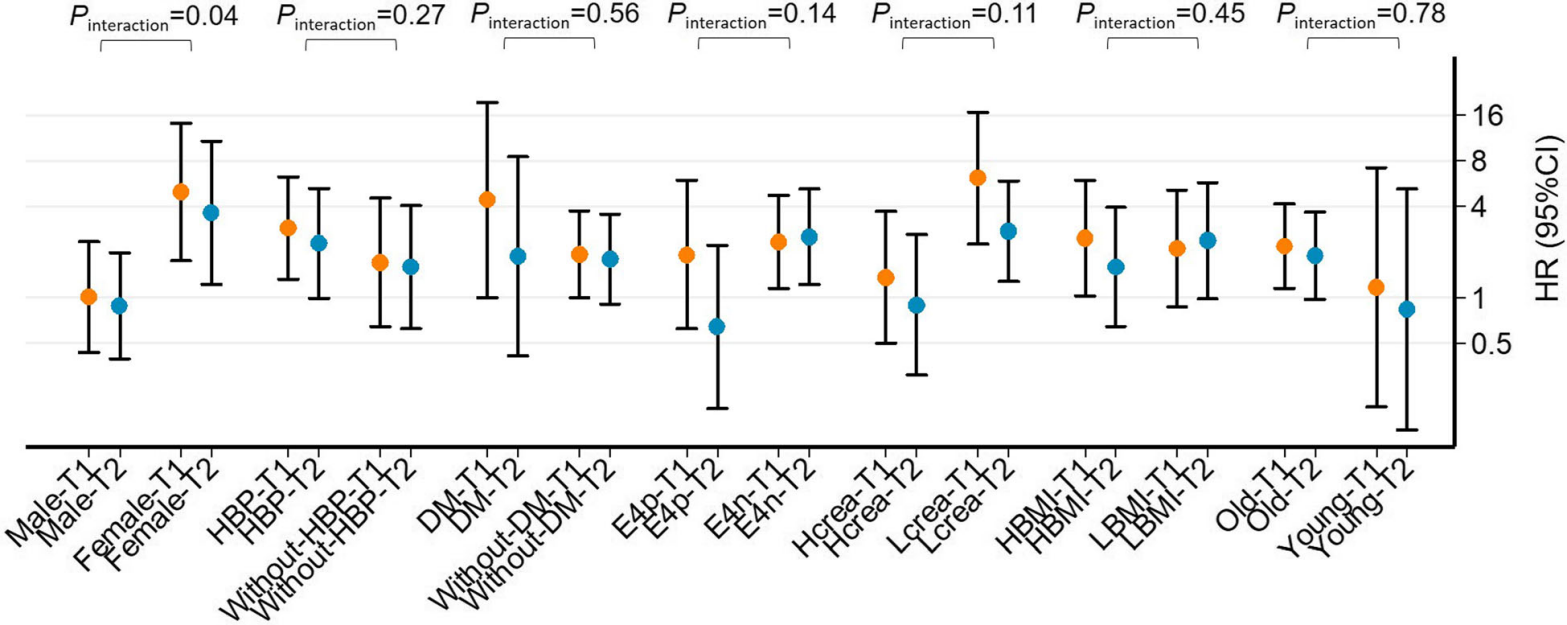
Subgroup analysis of the association of low or moderate glomerular filtration rate (GFR_cycys_) versus high GFR_cycys_ with onset of dementia in fully adjusted model in the Shanghai aging population. Points and error bars represent point estimates and 95% confidence intervals, respectively. T1 (low GFR, Tertile1), GFR_cycys_ < 67 ml/min/1.73 m^2^; T2 (moderate GFR, Tertile2), 67 < =GFR_cycys_ < 80 ml/min/1.73 m^2^; T3 (high GFR, Tertile3), GFR_cycys_ > =80 ml/min/1.73 m^2^; HBP, hypertension; DM, diabetes; H_crea_, serum creatinine ≥ 75 μmol/l; L_crea_, serum creatinine < 75 μmol/l; E4p, E4 positive; E4n, E4 negative; HBMI, BMI ≥ 24 kg/m^2^; LBMI, BMI < 24 kg/m^2^; Old, age ≥ 70 years old; Young, age < 70 years old for interaction terms. *P*_interaction_ was the result of interaction effect

As shown in Fig. 2, the associations of low and middle GFR_crcys_ (reference: high GFR_crcys_) with incident dementia were examined in subgroups by multivariate Cox model. Among female participants, low GFR_crcys_ (adjusted HR = 4.98 [95% CI 1.75 to 14.17]) and moderate GFR_crcys_ (adjusted HR = 3.63 [95% CI 1.22 to 10.81]) were associated with higher risk of incident dementia, while no association was found in male participants. The interaction was significant in gender (*P*_interaction_ = 0.04). However, the association between GFR and dementia was not modified by age (≥ 70 or < 70 years old), baseline diabetes, hypertension, APOE-ε4, serum creatinine (≥ 0.85 or < 0.85 mg/dl), and BMI (≥ 24 or < 24 kg/m^2^) (*P*_interaction_ > 0.05 for all). Since the association between GFR and dementia was modified by gender, we also included the interaction term of gender×GFR as a confounder into the original fully adjusted model. Finally, we found that low and moderate GFR still increased the risk of dementia and AD (Table S3).

## Discussion

In the present study, we provided evidence that lower baseline GFR_crcys_ is associated with higher risk of all-cause incident dementia and AD in a Chinese community-dwelling sample of older adults after controlling for confounders. In addition, the association was found significantly in women but not in men. Besides the accurate estimation of GFR as GFR_crcys_ for the kidney function, strengths of the current study include the prospective study design, a relatively large sample size of cohort particularly focus on cognitive impairment, and adjustment of important confounders including baseline MMSE score and APOE-ε4. The diagnosis of cognitive function was based on complete clinical, neuropsychological assessments, and consensus diagnosis for each participant both at the baseline and follow-up interview.

A few studies reported that low GFR or late CKD stages were associated with impairment of global cognition [24–26], executive function [27–29], processing speed [30], and episodic memory [31] in community-dwelling older adults. Some prospective studies also reported the association between renal function and incident dementia in community-dwelling adults but the results are divergent. The Cardiovascular Health Cognition Study (CHCS) with 3349 participants over the age of 65 reported that every 0.5-unit decrement in 1/SCr was associated with a 26% increased risk of incident dementia; besides, higher SCr was associated with vascular dementia but not AD [8]. In the Three-City (3C) study, results showed that an initial low GFR_cr_ (estimated by creatinine-based CKD-EPI equation) was not associated with increased risk of incident dementia. However, the decline in GFR_cr_ was associated with all-cause dementia and vascular dementia, but not AD [7]. In the Adult Changes in Thought (ACT) study with 2968 community-based individuals aged over 65 years enrolled, participants with greater variability in GFR_cr_ but not low baseline GFR showed a higher incidence of dementia [6]. The Rotterdam Study followed up 5993 participants without dementia or previous stroke at baseline for a mean of 11.6 years. It showed that lower GFR_crcys_ was related to a higher risk of stroke but not dementia, especially in those aged ≥ 70 years [9]. By using the data of the Atherosclerosis Risk in Communities (ARIC) study, Scheppach et al. found lower eGFR based on cystatin-C or beta-2-microglobulin, but not creatinine, which was also associated with dementia [10]. Only one study was conducted in Asian; however, Keita Takae did not find any association between GFR and incident dementia in 1562 community-dwelling Japanese subjects [11].

Despite the target population, sample size, and follow-up time, the inconsistent conclusion from these studies may be due to the measurement of kidney function. The earliest CHCS study used indirect markers of renal function, such as serum creatinine, while the ACT study, Hisayama study, and 3C study used creatinine only-based CKD-EPI equation. In the current study, we used the combination of serum creatinine and serum cystatin C CKD-EPI equation to assess GFR. This measurement was derived in 2012 and was demonstrated to permit more accurate GFR estimation and more precise risk prediction for adverse outcomes than any other GFR estimation equations [32–34]. We also did the analysis by using creatinine alone-based CKD-EPI equation, but did not obtain the significant findings (Table S1). This may demonstrate the importance of including cystatin C when estimating GFR for elderly individuals whose serum creatinine can be affected by lean muscle mass [13]. Though using the same measurement as GFR_crcys_, the Rotterdam study did not achieve similar findings as ours. Because in the statistical analysis, the censoring was applied to the risk of stroke and dementia separately [9]. A close link between stroke and dementia in terms of etiology and their frequent co-occurrence could be the explanation [9]. In our study, we did a sensitivity analysis in participants without stroke history, and the association was still significant. Different from the ARIC study [10], the prediction of GFR_crcys_ to cognition impairment not only includes dementia but also AD, the most common form of dementia manifested as an irreversible and progressive course of disease and the cause of which is still poorly understood; besides, the treatment is far from satisfactory. In addition to a consistent diagnosis of dementia by a multidisciplinary team, more importantly, the associations in the current study were adjusted for baseline global assessment of cognitive status by MMSE, and this may eliminate the attribution of incident dementia to baseline cognitive impairment in those with lower baseline GFR. Nevertheless, the nature of the cohort, the sample size, the confounders chose in the models, and the statistical method using to ensure the comparability between groups all influence the conclusion we obtained. Future studies are still needed to verify current finding.

Several potential mechanisms may help to explain the association between reduced GFR and dementia. Small arterial changes are crucial primary contributors to the development of kidney aging, which is the main reason for GFR decline in our community-dwelling older adults with a low prevalence of proteinuria [35]. It is manifested by increased extracellular matrix deposition, increased intimal cell proliferation, increased intrarenal shunting, capillary bypassing, and an imbalance of endothelial cell-derived factors altering vascular tone and vasomotor activity [35–37]. The brain and the kidneys have many common anatomic and vasoregulatory features [38]. The above-mentioned loss of endothelial dysfunction and vascular integrity could partly explain the vasculopathy-related dementia [39]. An animal study with molecular imaging also explored that the extent of abnormal endothelial activation in aged mice was more severe in the kidney than in the brain [40]. Nonvascular risk factors might further contribute to dementia. In our study, we obtained an independent association between low GFR and dementia even after excluding participants with stroke history. Kidney function decline accompanies with lower serum α-Klotho and vitamin D, higher cystatin-C, and homocysteine level. A reduced blood α-Klotho level is correlated with grading of cerebral deep white matter lesions, while lower serum vitamin D concentration is associated with amyloid-β formation [41, 42]. Cystatin-C as an inhibitor of cysteine proteases may have a direct effect on the risk of developing AD [43]. Hyperhomocysteinemia has direct neurotoxicity through the overstimulation of N-methyl-D-aspartate receptors; therefore, it is associated with ischemic leukoaraiosis and subsequent dementia [44, 45]. Furthermore, it is hypothesized that circulating levels of inflammatory molecules elevating in low GFR of aging individuals may cross the blood-brain barrier to interact with neurotrophic factors and with reactive species of oxygen and thus contribute to neuropsychiatric disorders [46].

In the subgroup analysis, we found that the association between GFR and dementia was modified by gender: women with lower GFR were more likely to develop dementia than men. This may because of the interaction of low GFR with estrogen. Due to conversion pathways from testosterone, older men have higher levels of estrogen than post-menopausal women [47]. As we mentioned above, lower GFR may be associated with amyloid-β formation while low estrogenic compounds aggravate mitochondria toxicity of amyloid-β [48]. Besides, a lower brain volume, less educational attainment and workforce, along with higher levels of frailty than men, all render women more susceptible to cognitive impairment under lower GFR [14, 49]. Some studies reported that concurrent presence of diabetes and impaired kidney function was associated with a substantial likelihood of cognitive impairment in older adults [50]. Other studies also provided firm neuroprotective effects by individual nutrients. However, we did not found any significant interaction of comorbidities, serum creatinine, or BMI with GFR decline [51]. Nevertheless, results need to be verified in future studies with longer follow-up.

### Limitations

There are some limitations in this study. First, quite a few participants with lost to follow-up may lead to selection bias. However, included participants were younger by averaged 2 years old and had higher BMI and MMSE score at baseline (Table S2); therefore, the association in our results might be underestimated. Second, the association of low GFR and incident dementia or AD was attenuated after controlling for various confounders, which indicates the association may be driven in part by the demographic and comorbid factors. Since decrease of kidney function is related with many aspects of physiological and pathological condition which might have influence on dementia, there might be other confounding variables related to kidney function but have not been measured, such as medications or other comorbidities. Therefore, larger sample size with more complex analysis may be done to further demonstrate current findings. Third, it would be better to use urine albumin to creatinine ratio (ACR) to identify kidney disease instead of urine routine test of protein since urine ACR has greater sensitivity for low levels of proteinuria and improves the detection of kidney disease in the study population [52]. Last, our study was limited to a cohort of Chinese individuals; therefore, our results may not be generalizable to populations with other races.

## Conclusions

This prospective study found that low GFR_crcys_ was independently associated with high risk of incident dementia and AD in community-dwelling older adults. GFR_crcys_ may be considered as a marker of an individual’s vulnerability to the increased risk of cognitive decline. More research is needed to examine whether the association with dementia is causal or due to shared mechanisms, and to explore the underlying pathophysiology.

## Supplementary Information

**Additional file 1.**

## Data Availability

The datasets used and/or analyzed during the current study are available from the corresponding author on reasonable request.

## Abbreviations

GFR: Glomerular filtration rate
CKD-EPI: Chronic Kidney Disease Epidemiology Collaboration
AD: Alzheimer’s disease
MCI: Mild cognitive impairment
APOE)-e4: Apolipoprotein
MMSE): Mini-Mental State Examination
CESD: Center for Epidemiologic Studies Depression Scale
ADL: Activities of daily living
BMI: Body mass index
HR: Hazard ratio
CI: Confidence interval
CHCS: Cardiovascular Health Cognition Study
3C study: Three-City
ACT study: Adult Changes in Thought
ARIC study: Atherosclerosis Risk in Communities
ACR: Albumin to creatinine ratio
