# Supplementary material for: Kidney function and dementia risk in community-dwelling older adults: the Shanghai Aging Study
Source: Alzheimers Res Ther. 2021 Jan 11;13:21. doi: 10.1186/s13195-020-00729-9 (PMC7798296; doi:10.1186/s13195-020-00729-9)

**SUPPLEMENTARY MATERIALS**

**Kidney function and dementia risk in** **community-dwelling older adults: the Shanghai Aging Study**

Authors:

**Mengjing Wang, MD, PhD^1,3*^;Ding Ding, MPH, PhD^2,3^; Qianhua Zhao, MD, PhD^2,3^; Wanqing Wu, MS^2^; Zhenxu Xiao, MS^2^; Xiaoniu Liang, MPH^2^; Jianfeng Luo, PhD^4,5^;and Jing Chen, MD, PhD^1,3^**

Affiliations:

*^1^Department of Nephrology, Huashan Hospital, Fudan University, Shanghai, China;*

*^2^Institute of Neurology, Huashan Hospital, Fudan University, Shanghai, China;*

*^3^National Clinical Research Center for Aging and Medicine, Huashan Hospital, Fudan University, Shanghai, China.*

***^4^*** *Department of Biostatistics, School of Public Health, Fudan University, Shanghai, China;*

***^5^*** *Key Laboratory of Public Health Safety of Ministry of Education (Fudan University), Shanghai, China*

**Table S1. Associations of baseline glomerular filtration rate (GFR_cr_ or GFR_cys_) estimated by the CKD-EPI equations with incidence of dementia and Alzheimer disease in participants without stroke history.**

|  | **Dementia** | | | | **Alzheimer's disease** | | | |
| --- | --- | --- | --- | --- | --- | --- | --- | --- |
|  | Unadjusted | | Adjusted | | Unadjusted | | Adjusted | |
|  | HR (95%CI) | *P* | HR (95%CI) | *P* | HR (95%CI) | *P* | HR (95%CI) | *P* |
| **All participants** | | | | | | | | |
| **Tertile of GFR_cys_** |  |  |  |  |  |  |  |  |
| GFR_cys_>77 | Ref |  | Ref |  | Ref |  | Ref |  |
| 63≤GFR_cys_<77 ml/min/1.73m^2^ | 3.66 (1.96 to 6.85) | <0.001 | 1.87 (0.98 to 3.56) | 0.06 | 4.50 (2.16 to 9.38) | <0.001 | 2.23 (1.05 to 4.75) | 0.04 |
| GFR_cys_<63 ml/min/1.73m^2^ | 5.59 (3.07 to 10.19) | <0.001 | 2.05 (1.08 to 3.89) | 0.03 | 5.51 (2.67 to 11.34) | <0.001 | 1.89 (0.88 to 4.09) | 0.10 |
| **Tertile of GFR_cr_** |  |  |  |  |  |  |  |  |
| GFR_cr_>84 ml/min/1.73m^2^ | Ref |  | Ref |  | Ref |  | Ref |  |
| 70≤GFR_cr_<84 ml/min/1.73m^2^ | 1.62 (0.96 to 2.74) | 0.07 | 1.32 (0.77 to 2.28) | 0.32 | 1.89 (1.04 to 3.41) | 0.04 | 1.40 (0.75 to 2.62) | 0.29 |
| GFR_cr_<70 ml/min/1.73m^2^ | 2.60 (1.59 to 4.23) | <0.001 | 1.35 (0.81 to 2.26) | 0.25 | 2.34 (1.31 to 4.16) | 0.004 | 1.09 (0.59 to 2.02) | 0.78 |
| **Participants without stroke** | | | | | | | | |
| **Tertile of GFR_cys_** |  |  |  |  |  |  |  |  |
| GFR_cys_>77 | Ref |  | Ref |  | Ref |  | Ref |  |
| 63≤GFR_cys_<77 ml/min/1.73m^2^ | 3.34 (1.68 to 6.65) | 0.001 | 1.62 (0.79 to 3.30) | 0.19 | 4.40 (1.91 to 10.15) | 0.001 | 2.13 (0.90 to 5.04) | 0.09 |
| GFR_cys_<63 ml/min/1.73m^2^ | 4.90 (2.52 to 9.54) | <0.001 | 1.86 (0.90 to 3.85) | 0.09 | 5.81 (2.56 to 13.21) | <0.001 | 2.11 (0.87 to 5.11) | 0.10 |
| **Tertile of GFR_cr_** |  |  |  |  |  |  |  |  |
| GFR_cr_>84 ml/min/1.73m^2^ | Ref |  | Ref |  | Ref |  | Ref |  |
| 70≤GFR_cr_<84 ml/min/1.73m^2^ | 1.99 (1.09 to 3.65) | 0.03 | 1.46 (0.78 to 2.75) | 0.24 | 2.43 (1.20 to 4.91) | 0.01 | 1.65 (0.79 to 3.46) | 0.18 |
| GFR_cr_<70 ml/min/1.73m^2^ | 2.57 (1.43 to 4.64) | 0.002 | 1.35 (0.73 to 2.52) | 0.34 | 2.80 (1.39 to 5.65) | 0.004 | 1.35 (0.65 to 2.84) | 0.42 |

Abbreviations: GFR_cys_ glomerular filtration rate estimated by the CKD-EPI cystatin C equation; GFR_cr_ glomerular filtration rate estimated by the CKD-EPI creatinine equation; HR, Hazard ratio; CI, confidence interval.

^*^*P* adjusted for age, gender, education years, APOE-ε4 positive, diabetes, hypertension, mini-mental state examination (MMSE), and proteinuria (positive, negative)

**Table S2. Baseline characteristics of 1412 included individuals and 1573 excluded individuals.**

| **Characteristics** | Excluded individuals N=1,573 | Missing data for excluded individuals | Included individuals N=1,412 | Missing data for included individuals | Standardized difference |
| --- | --- | --- | --- | --- | --- |
| **Age (years)** | 72.87±8.42 | 0 | 70.69±6.85 | 0 | 0.28 |
| **Male(%)** | 722 (45.9%) | 0 | 659 (46.67%) | 0 | -0.02 |
| **Body mass index (kg/m2)** | 24.01±3.42 | 0.45% | 24.83±3.45 | 0.14% | -0.24 |
| **Education years** | 11.65±4.44 | 0 | 11.99±3.97 | 0 | -0.08 |
| **Smoking (%)** | 148 (9.46%) | 0.51% | 144 (10.22%) | 0.21% | -0.02 |
| **Comorbidities (%)** |  |  |  |  |  |
| Diabetes | 237 (15.11%) | 0.25% | 197 (13.95%) | 0 | 0.03 |
| Hypertension | 911 (58.06%) | 0.25% | 748 (52.97%) | 0 | 0.100 |
| Stroke | 210 (13.44%) | 0.64% | 180 (12.75%) | 0 | 0.02 |
| **MMSE** | 27.77±2.43 | 0 | 28.35±1.88 | 0 | -0.27 |
| **SBP (mmHg)** | 145.91±23.29 | 0.38% | 145.67±22.29 | 0.14% | 0.01 |
| **DBP (mmHg)** | 75.99±14.26 | 0.38% | 77.81±11.42 | 0.14% | -0.14 |
| **GFRcrcys (ml/min/1.73m^2^)** | 71.63±17.16 | 25.94% | 73.79±15.63 | 0 | -0.13 |
| **Proteinuria positive (%)** | 46(3.4%) | 13.99% | 37(2.63%) | 0.28% | 0.05 |
| **APOE-e4 allele positive (%)** | 246 (18.74%) | 16.52% | 233 (16.50%) | 0 | 0.06 |
| **Laboratory variables** |  |  |  |  |  |
| Triglyceride (mg/dL) | 149.58±85.90 | 14.18% | 156.56±102.45 | 0 | -0.07 |
| Cholesterol (mg/dL) | 205.56±39.48 | 14.11% | 208.55±40.67 | 0 | -0.08 |
| Low-density lipoprotein cholesterol (mg/dL) | 124.66±34.18 | 14.30% | 129.43±35.36 | 0 | -0.13 |
| High-density lipoprotein cholesterol (mg/dL) | 51.52±14.20 | 14.30% | 51.52±13.03 | 0 | 0 |

*Note*: Values are expressed as mean±SD or percentage, appropriately. Abbreviations: MMSE, mini-mental state examination. SBP, systolic blood pressure. DBP, diastolic blood pressure. APOE, apolipoprotein E.

**Table S3. Associations of baseline glomerular filtration rate (GFR**_crcys_**) with incidence of dementia and Alzheimer disease in the fully adjusted model including the interaction term of gender×GFR.**

|  | **Dementia** | | **Alzheimer's disease** | |
| --- | --- | --- | --- | --- |
|  | Adjusted | | Adjusted | |
|  | HR (95%CI) | *P^a^* | HR (95%CI) | *P^a^* |
| All participants |  |  |  |  |
| **Tertile of GFR_crcys_** |  |  |  |  |
| GFR_crcys_>80 ml/min/1.73m^2^ | Ref |  | Ref |  |
| 67≤GFR_crcys_<80 ml/min/1.73m^2^ | 4.19 (1.42 to 12.38) | 0.01 | 4.24 (1.23 to 14.63) | 0.022 |
| GFR_crcys_<67 ml/min/1.73m^2^ | 5.28 (1.87 to 14.90) | 0.002 | 4.39 (1.32 to 14.68) | 0.016 |
| Participants without stroke |  |  |  |  |
| **Tertile of GFR_crcys_** |  |  |  |  |
| GFR_crcys_>80 ml/min/1.73m^2^ | Ref |  | Ref |  |
| 67≤GFR_crcys_<80 ml/min/1.73m^2^ | 4.16 (1.18 to 14.64) | 0.027 | 4.83 (1.07 to 21.87) | 0.041 |
| GFR_crcys_<67 ml/min/1.73m^2^ | 6.30 (1.90 to 20.87) | 0.003 | 6.25 (1.44 to 27.03) | 0.014 |
|  |  |  |  |  |

Abbreviations: GFR_crcys_, glomerular filtration rate estimated by the CKD-EPI creatinine–cystatin C equation; HR, Hazard ratio; CI, confidence interval.

^a^*P* adjusted for age, gender, education years, APOE-ε4 positive, diabetes, hypertension, mini-mental state examination (MMSE), proteinuria (positive, negative), and gender×GFR category.

**Fig S1.** Cohort construction.


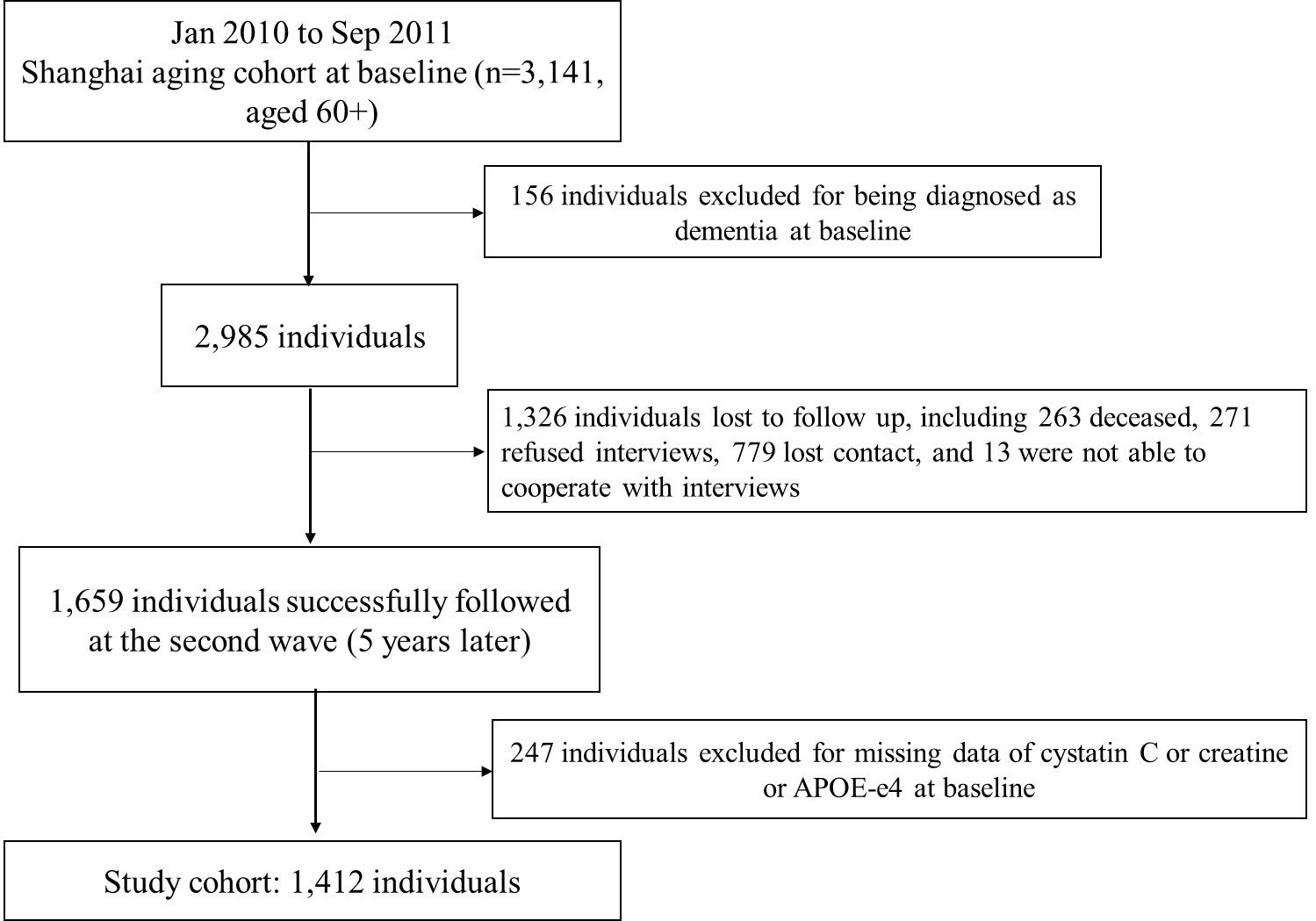

Supplement: Supplementary file 1 — Additional file 1. [file 13195_2020_729_MOESM1_ESM.docx]
